# Understanding excess mortality during COVID in Belgium: the influence of pre-existing health status and social factors

**DOI:** 10.1186/s13690-025-01499-2

**Published:** 2025-01-23

**Authors:** Laura Van den Borre, Brecht Devleesschauwer, Sylvie Gadeyne, Katrien Vanthomme, Didier Willaert

**Affiliations:** 1 Department of Epidemiology and Public Health, Sciensano, Brussels, Belgium; 2 Department of Translational Physiology, Infectiology and Public health, Ghent University, Merelbeke, Belgium; 3 Brussels Institute for Social and Population Studies, Vrije Universiteit Brussel, Brussels, Belgium; 4 Department of Public Health and Primary Care, Ghent University, Ghent, Belgium; 5 Solidaris - Socialist Health Insurance Fund, Brussels, Belgium

**Keywords:** Multimorbidity, Chronic diseases, Socio-economic status, COVID-19, All-cause mortality

## Abstract

**Background:**

This study aims to investigate how pre-existing health status and social background contribute to excess mortality during the COVID-19 crisis in Belgium.

**Methods:**

The study population consists of almost 1.4 million adult members of Solidaris, the second largest health insurance fund in Belgium. Pre-existing health status was identified using health care reimbursement data, including medication use. Social characteristics included a proxy for low socio-economic status, nationality of origin, and living arrangement. Excess mortality during the COVID-19 crisis was measured by computing the relative difference between all-cause mortality in 2020 or 2021 and the average yearly mortality in 2015–2019. Directly Standardised Mortality Rates (DSMRs) were calculated to investigate absolute mortality inequalities. Mortality Rate Ratios (MRRs) were computed using Poisson regression analyses to investigate relative mortality inequalities.

**Results:**

DSMRs show that persons with no previous disease experienced significant excess mortality in 2020, like men with one, two or three diseases and women with various numbers of pre-existing diseases. Results by specific disease show heterogenous results. After adjusting for age, sex and social characteristics, persons with cancer experienced a significant mortality deficit of 17% in 2020 and of 9% in 2021. For persons with cancer and asthma or COPD, significant mortality deficits of 10% and  3% were observed in 2020 and 2021, respectively.

**Conclusion:**

The study provides insights into the complex dynamics of mortality during the COVID-19 crisis, emphasising the need to consider individual-level information on pre-existing health and social background jointly.

**Supplementary Information:**

The online version contains supplementary material available at 10.1186/s13690-025-01499-2.

**Table:** 

**Text box 1. Contributions to the literature**	
• There is a lack of literature on excess mortality during the COVID-19 crisis that investigates pre-existing health, sociodemographic and socioeconomic factors on the individual level jointly.	
• The heterogeneous results by specific disease group underline the need to account for pre-existing health situation.	
• Results suggest a potential for underestimation and overestimation of mortality rates by diseases group when not considering sociodemographic and socioeconomic factors.	

## Introduction

The COVID-19 crisis has placed an unequal burden on population health. Although the syndemic nature of the crisis is not yet fully understood, recent research indicates that the COVID-19 pandemic interacts with and is exacerbated by pre-existing social, economic and health inequalities [1]. Crisis-specific risk factors and comorbidities are expected to be intertwined and have an interactive and cumulative effect on population health. COVID-19 severity has been clearly associated with advanced age and male sex [2, 3]. Social inequalities in COVID-19-related health outcomes have been reported with higher risks for exposure, infection, symptom severity, hospitalisation and death among disadvantaged groups (e.g., low-income groups, people with a migrant background) and specific occupations (e.g., health care workers) [4–7].

In addition to the direct effects of the outbreak, the crisis also has indirect health effects including economic hardship, social isolation, psychological distress, and delayed healthcare-seeking behaviour [8–11]. In order to prevent the healthcare system from being submerged by an overwhelming number of COVID-19 patients, regular health care in Belgium was suspended from March 14th 2020 until May 4th 2020 [12]. All non-urgent and selective primary and hospital care was postponed reserving maximum capacity for triage and hospitalisation of COVID patients. This postponement is likely to have unintended consequences on access to care, diagnosis, and treatment. Despite the resumption of the regular health care and substantial catch-up initiatives, an audit of the Belgian hospital capacity shows persistent disruptive effects during recent COVID-19 waves [13]. As a result of these direct and indirect crisis effects, persons with pre-existing health issues are expected to have experienced poorer health and increased mortality risks during the COVID-19 crisis, especially those from socially disadvantaged groups.

To quantify the overall mortality impact of the crisis, previous studies have monitored excess mortality. The comprehensive metric builds on the pre-pandemic mortality patterns to compare the observed number of deaths [14, 15]. Excess mortality also includes the indirect effects of the crisis and is therefore not affected by underdiagnosis and underreporting of specific causes of death. Considerable evidence is found for significant social differences in excess mortality in various national contexts [e.g., 16–20]. However, to our knowledge, few studies investigated the joint role of pre-existing health conditions and social factors on population mortality [21, 22]. In addition, most available research draws on data from patient cohorts [23]. These results may not be representative for the general population and the representativeness for all patient profiles is highly dependent on the cohort inclusion criteria. Several population mortality studies have investigated aggregate information on the health context and social background [24, 25]. Although this approach is useful to explore potential health and social inequalities at the area level, individual-level data are needed to examine the interplay of health and social factors on population mortality [26].

The current study sets out to better understand the drivers of excess mortality during the COVID-19 crisis using individual-level information from health insurance fund Solidaris. To this end, our main objective is to estimate excess mortality by pre-existing health status and social background characteristics.

## Method

The study population includes approximately 1.4 million individuals, aged 40 years and older, who are member of the health insurance fund Solidaris (Supplementary table S1). Health insurance is mandatory in Belgium and organised by the public sector. From the seven Belgian health insurance funds, Solidaris is the second largest, covering approximately 28.5% of the total population eligible for reimbursement in Belgium (e.g., Belgian population, Eurocrats). Health insurance funds collect and manage all data on health care expenditures as well as information on reimbursed medicines in pharmacies. Because information on pre-existing diseases is not available, a proxy was developed by an expert group of the National Institute for Health and Disability Insurance (RIZIV-INAMI). Anatomical, therapeutic, and chemical (ATC) codes for the prescribed and reimbursed medication from pharmacies were used to identify a number of chronic health conditions. To increase the accuracy of assigning ATC codes to a condition, the ATC codes are used in combination with a minimum threshold of 90 Defined Daily Dose per year and/or the age of the individual for specific diseases. The methodology behind the algorithm to construct these specific proxies or “pseudo-pathologies” has been described by the Intermutualistic Agency [27]. Although this data source provides essential information on healthcare consumption and health expenditures, it lacks information on the use of non-reimbursed health care services and on health needs related to undiagnosed or untreated conditions.

Pre-existing health status is determined in two steps. First, it considers the presence of six specific conditions: asthma or chronic obstructive pulmonary disease (COPD), cancer, cardiovascular diseases (CVD), chronic kidney disease, diabetes, and anxiety or depression. These conditions were chosen from the list of defined proxies because of their prevalence in Belgium and previously established associations with severe COVID-19 outcomes, which may entail increased mortality risks. Diagnoses with chronic kidney disease, cardiovascular diseases and diabetes were associated with confirmed COVID-19 infections in Flanders during the first two COVID-waves [28]. In addition, these conditions have been identified, along with cancer and chronic lung diseases, as potential risk factors for severe COVID-19 outcomes [29–31]. Furthermore, an international systematic review revealed associations between COVID-19 mortality and pre-existing mental disorders and exposure to antipsychotics and anxiolytics [32]. The operationalisation of these specific proxies is based on information on reimbursed and prescribed medicines in the previous year (Supplementary table S2), as documented by the Intermutualistic Agency [27]. Second, the developed health conditions proxies were used to determine the number of comorbidities per individual in each previous year.

The Solidaris database also includes information on the sociodemographic background. Age in years was calculated on January 1st of each study year, building on the registered birth date. Sex was available in a binary categorisation (male/female); region of residence was derived from the municipality of residence. Following categories of living arrangements were considered: persons living with a partner, in one-person households, other living arrangements, and care home residency. Nationality of origin was operationalised using information on current nationality, historical information on nationality since 2001, and the lineage of the member. Three categories were discerned: Belgian; Western migrant (European countries, United States, Canada, Japan, Australia, New-Zealand) and Non-Western migrant (all other countries).

To gauge the association with the socioeconomic context, information on whether a person received increased reimbursement was included. The statute for increased reimbursement is granted to individuals or families with a low income and/or who are receiving social benefits (e.g., increased support for elderly persons, or an allowance for disabled persons granted by the Federal Public Service Social Security) [33].

All-cause mortality information was included from January 1st, 2015 to December 31st, 2021. Annual cohorts were created to investigate the mortality pattern during each study year in combination with the health and social profile. Because of the relative low mortality rates among younger age groups, analyses were restricted to adults aged 40 and older. Excess mortality during the COVID-19 crisis was measured as the relative difference between all-cause mortality in 2020 or 2021 and the geometric mean of the yearly mortality estimates in 2015–2019 [34]. The geometric mean of the yearly estimates for 2015–2019 was calculated to create a robust summary estimate for the reference period [34]. Inequality in excess mortality was investigated in two ways. First, Directly Standardised Mortality Rates (DSMR) were calculated for each study year using the total Belgian population on January 1st, 2021 as standard population to investigate absolute mortality inequalities. Second, Mortality Rate Ratios (MRR) were estimated using Poisson regression analyses to study relative mortality inequalities. The statistical significance of the absolute and the relative change in mortality over time was formally tested as explained by Altman and Bland [35]. In order to investigate the relative importance of pre-existing health, sociodemographic and socioeconomic characteristics, models were built using block-wise selection. The first model incorporated the indicator for pre-existing health status, age and sex. In the second model the sociodemographic characteristics – namely, region of residence, living arrangement, and nationality of origin- were added. The final model further introduced the indicator for increased reimbursement. Analyses were performed using SAS (version 9.4) and STATA (version 16).

## Results

### Absolute mortality inequalities

DSMRs were calculated by sex, pre-existing health status and social background characteristics (Supplementary table S3). Men experienced 1,486 deaths per 100,000 representing an excess mortality of 10% in 2020 compared to the reference period 2015–2019. The absolute mortality burden among women was somewhat lower compared to men with 1,031 deaths per 100,000. Mortality among women was 12% higher than in the reference period. The mortality pattern in 2021 revealed that men experienced similar mortality levels in 2021 compared to the reference period (DSMR 1,350 deaths per 100,000), while women experienced a small but significant mortality deficit of 3.5% (DSMR 887 deaths per 100,000).

The results by pre-existing health status in Table 1 show that both individuals with poor pre-existing health and those with no registered previous disease experienced a significant mortality increase in 2020. Significant excess mortality was found for men with one, two, or three diseases, who experienced between 9.5% and 7.7% more deaths in 2020 compared to the reference period. The absence of a disease in men was associated with 7.9% more deaths in 2020 compared with the reference period. Results for men with four or more diseases were not significant. For women, all categories of number of diseases showed significant excess mortality ranging between 7.4% for women with two diseases and 24.4% for women with five or more diseases. Women with no diseases experienced 10.7% more deaths in 2020 compared to the reference period. The 2021 mortality patterns revealed significant results for four specific subgroups, but interestingly, these results indicate a mortality deficit. Mortality in 2021 was 7.3% and 6.4% lower for men and women with three diseases compared to the reference period. Women with no diseases and with two diseases had a mortality deficit of 8.5% and 4.2%, respectively.

Results for specific disease proxies indicated that men and women with cardiovascular disease experienced 11.0% and 12.8% higher mortality in 2020, respectively, compared to the reference period. Male and female diabetes patients experienced a 16.1% and 14.0% mortality increase in 2020 compared to the reference period. Anxiety or depression was associated with an excess mortality of 11.5% among men and of 15.7% among women in 2020. In contrast, in 2021, anxiety or depression was associated with a mortality deficit of 6.3% among men and 5.4% among women in 2021. For male cancer patients, a significant mortality deficit of 9.2% was observed in 2020 and of 15.2% in 2021. For women, the deficit for cancer patients was only apparent in the 2021 results (-8.9%). In addition, men and women with asthma or COPD experienced 5% and 12.2% fewer deaths in 2021 compared to the reference period.

**Table 1:** Directly standardised mortality rates (DSMR) per 100,000 person-years and relative differences (2015–2019) by pre-existing health status

	2015–2019			2020			2021			
	DSMR /100,000	CI		DSMR /100,000	CI	Rel.diff.	DSMR /100,000		CI	Rel.diff.
**A) Men**										
**Number of comorbidities**										
**No disease**	993	(943–1043)		1071	(1021–1122)	7.9%*	998		(949–1047)	0.5%
**1 disease**	1213	(1172–1254)		1329	(1290–1367)	9.5%*	1224		(1186–1261)	0.9%
**2 diseases**	2004	(1937–2070)		2171	(2109–2233)	8.3%*		1931	(1872–1990)	-3.6%
**3 diseases**	3168	(3026–3310)		3410	(3279–3542)	7.7%*		2938	(2819–3056)	-7.3%*
**4 diseases**	4398	(4113–4683)		4803	(4514–5091)	9.2%		4167	(3892–4442)	-5.3%
**5 diseases or more**	6151	(5436–6866)		5694	(5116–6271)	-7.4%		5754	(5110–6398)	-6.5%
**Disease**										
**No disease**	993	(943–1043)		1071	(1021–1122)	7.9%*		998	(949–1047)	0.5%
**Cancer**	8092	(7716–8467)		7345	(7001–7689)	-9.2%*		6863	(6548–7178)	-15.2%*
**Kidney disease**	2767	(2353–3180)		3162	(2814–3509)	14.3%		2760	(2402–3118)	-0.3%
**Asthma/COPD**	2924	(2818–3030)		2964	(2868–3059)	1.4%		2778	(2685–2871)	-5.0%*
**Diabetes**	1923	(1832–2014)		2234	(2153–2314)	16.1%*		2012	(1931–2093)	4.6%
**CVD**	1681	(1645–1717)		1865	(1831–1900)	11.0%*		1698	(1666–1731)	1.0%
**Anxiety/Depression**	2788	(2684–2891)		3109	(3007–3211)	11.5%*		2611	(2516–2706)	-6.3%*
**B) Women**										
**Number of comorbidities**										
**No disease**	738	(701–774)		816	(780–853)	10.7%*		675	(642–709)	-8.5%*
**1 disease**	814	(790–837)		918	(893–942)	12.8%*		787	(764–810)	-3.2%
**2 diseases**	1272	(1236–1309)		1367	(1331–1403)	7.4%*		1219	(1185–1254)	-4.2%*
**3 diseases**	1911	(1837–1984)		2090	(2018–2161)	9.4%*		1788	(1719–1857)	-6.4%*
**4 diseases**	2652	(2484–2820)		2924	(2763–3085)	10.3%*		2444	(2298–2591)	-7.8%
**5 diseases or more**	3519	(3129–3909)		4379	(3976–4782)	24.4%*		3180	(2837–3523)	-9.6%
**Disease**										
**No disease**	738	(701–774)		816	(780–853)	10.7%*		675	(642–709)	-8.5%*
**Cancer**	5809	(5557–6062)		5801	(5567–6036)	-0.1%		5293	(5063–5523)	-8.9%*
**Kidney disease**	2057	(1648–2464)		2375	(2048–2701)	15.5%		2415	(2048–2782)	17.4%
**Asthma/COPD**	2071	(1996–2146)		2051	(1982–2119)	-1.0%		1818	(1752–1883)	-12.2%*
**Diabetes**	1424	(1367–1480)		1622	(1567–1678)	14.0%*		1386	(1333–1440)	-2.6%
**CVD**	1158	(1137–1180)		1307	(1284–1329)	12.8%*		1128	(1107–1149)	-2.6%
**Anxiety/Depression**	1591	(1547–1634)		1840	(1795–1884)	15.7%*		1505	(1464–1546)	-5.4%*

Note: Reference population: total Belgian population on January 1st, 2021. Results stratified by sex; Abbreviation: (*) significant difference at *p* = 0.05 with 2015–2019 DSMRs

Almost all sociodemographic and socioeconomic indicators under investigation were associated with significant excess mortality in 2020 (Supplementary table S3). The highest significant excesses were observed among persons with increased reimbursement (men: 12.8%; women: 16.1%); care home residents (men: 23.5%; women: 16.2%); migrants of non-western descent (men: 47.5%; women: 59.5%); and residents of the Brussels-Capital Region (men: 21.8%; women: 25.0%). The 2021 mortality pattern for men only showed a significant excess of 8.2% for men living with a partner. For women, significant mortality deficits were observed for receiving no increased reimbursement (-3.2%), for one-person households (-4.1%), for persons of Belgian descent (-4.7%), and for Flemish (-5.1%) and Walloon (-3.9%) residents. One notable exception are the results for migrant women of non-western descent, who experienced 39.8% more deaths in 2021 than expected from the reference period.

### Relative mortality inequalities: number of comorbidities

Relative mortality inequalities were gauged using Poisson regression analyses. Table 2 presents the results from the final model with adjustment for the number of diseases and social background characteristics. A clear stepwise gradient is found for the number of diseases, with persons suffering from five or more illnesses experiencing more than 4.5 times higher mortality than persons with no disease in all study periods. As presented in supplementary table S4, the MRRs for persons with one or two diseases remained stable after including the social background characteristics in the model. In contrast, for persons with three, four or five or more diseases, MRRs demonstrated a strong decline after accounting for social background characteristics. For example, persons with 5 or more diseases had an age-and sex-adjusted MRR of 6.33 (CI 5.91–6.79) in 2020, which decreased markedly to a fully adjusted MRR of 4.58 (CI 4.24–4.94).

There is no indication of widening mortality inequalities for those receiving increased reimbursement, who experienced 31–35% higher mortality in all study periods. However, we did observe some differences with the pre-pandemic period. Compared to persons living with a partner, care home residents had a relatively high MRR of 4.82 (CI 4.49–5.18) in 2015–2019; a slightly higher MRR of 5.05 (CI 4.88–5.24) in 2020 and a significantly reduced MRR of 3.77 (CI 3.63–3.91) in 2021. A drop in MRRs for one-person households (compared to persons living with a partner) was found with 66% higher mortality in 2015–2019 versus approximately 55% higher mortality in 2020 and 2021. In addition, persons from non-Western origin consistently experienced lower mortality compared to native Belgians. This migrant mortality advantage shrank significantly during COVID times with people originating from non-Western countries experiencing 46% lower mortality in 2015–2019 and 28% lower mortality in 2020. Although still significantly different from the reference period, the results for 2021 indicate a readjustment back to the pre-pandemic situation, with 34% lower mortality observed among persons from non-Western migrants. Finally, regional mortality differences significantly widened. Compared with Solidaris members living in Flanders, Walloon residents experienced 9% higher mortality in 2015–2019, 19% higher mortality in 2020 and 13% higher mortality in 2021, compared with Flemish residents. There is also evidence of a widening mortality gap between Brussels residents and Flemish with 13% higher risk for Brussels residents in 2020, but this difference is no longer significant in 2021.

**Table 2:** Mortality rate ratios (MRR) and 95% confidence intervals (CI) by study period, final model

	2015–2019		2020		2021		
	MRR	(CI)	MRR	(CI)		MRR	(CI)
**Number of comorbidities**							
**No disease (ref)**	1.00		1.00			1.00	
**1 disease**	1.33	(1.29–1.36)	1.32	(1.27–1.38)		1.35	(1.30–1.41)
**2 diseases**	2.00	(1.93–2.08)	1.94	(1.86–2.02)		2.03	(1.94–2.12)
**3 diseases**	2.83	(2.74–2.92)	2.72	(2.60–2.84)		2.82	(2.69–2.96)
**4 diseases**	3.69	(3.51–3.88)	3.61	(3.42–3.81)		3.73	(3.52–3.95)
**5 diseases or more**	4.58	(4.29–4.89)	4.58	(4.24–4.94)		5.04	(4.64–5.47)
**Age (continuous)**	1.08	(1.08–1.08)	1.08	(1.08–1.08)		1.08	(1.08–1.08)
**Sex**							
**Women (ref)**	1.00		1.00			1.00	
**Men**	2.05	(2.00-2.10)	2.04	(1.99–2.08)		2.08	(2.03–2.13)
**Living arrangement**							
**Living with partner (ref)**	1.00		1.00			1.00	
**One-person household**	1.66	(1.57–1.75)	1.56	(1.51–1.61)		1.54*	(1.49–1.59)
**Other**	3.88	(3.05–4.94)	3.19	(3.03–3.36)		3.21	(3.04–3.39)
**Care home resident**	4.82	(4.49–5.18)	5.05	(4.88–5.24)		3.77*	(3.63–3.91)
**Nationality of origin**							
**Belgian (ref)**	1.00		1.00			1.00	
**Western**	0.86	(0.82–0.90)	0.88	(0.84–0.93)		0.89	(0.84–0.94)
**Non-western**	0.54	(0.51–0.57)	0.72*	(0.67–0.78)		0.66*	(0.61–0.72)
**Region of residence**							
**Flanders (ref)**	1.00		1.00			1.00	
**Brussels-Capital Region**	0.98	(0.96-1.00)	1.13*	(1.08–1.17)		1.03	(0.99–1.08)
**Wallonia**	1.09	(1.08–1.10)	1.19*	(1.16–1.22)		1.13*	(1.11–1.16)
**Increased reimbursement**							
**No (ref)**	1.00		1.00			1.00	
**Yes**	1.31	(1.29–1.34)	1.35	(1.32–1.38)		1.33	(1.30–1.37)
**Pseudo R-squared**	0,19^+^		0.21			0.18	
**BIC**	258.031^+^		289.108			272.485	

Note: ^**+**^ Average of the annual models; * significant difference at *p* = 0.05 with 2015–2019 MRRs

### Relative mortality inequalities: specific disease background

**Fig. 1:**
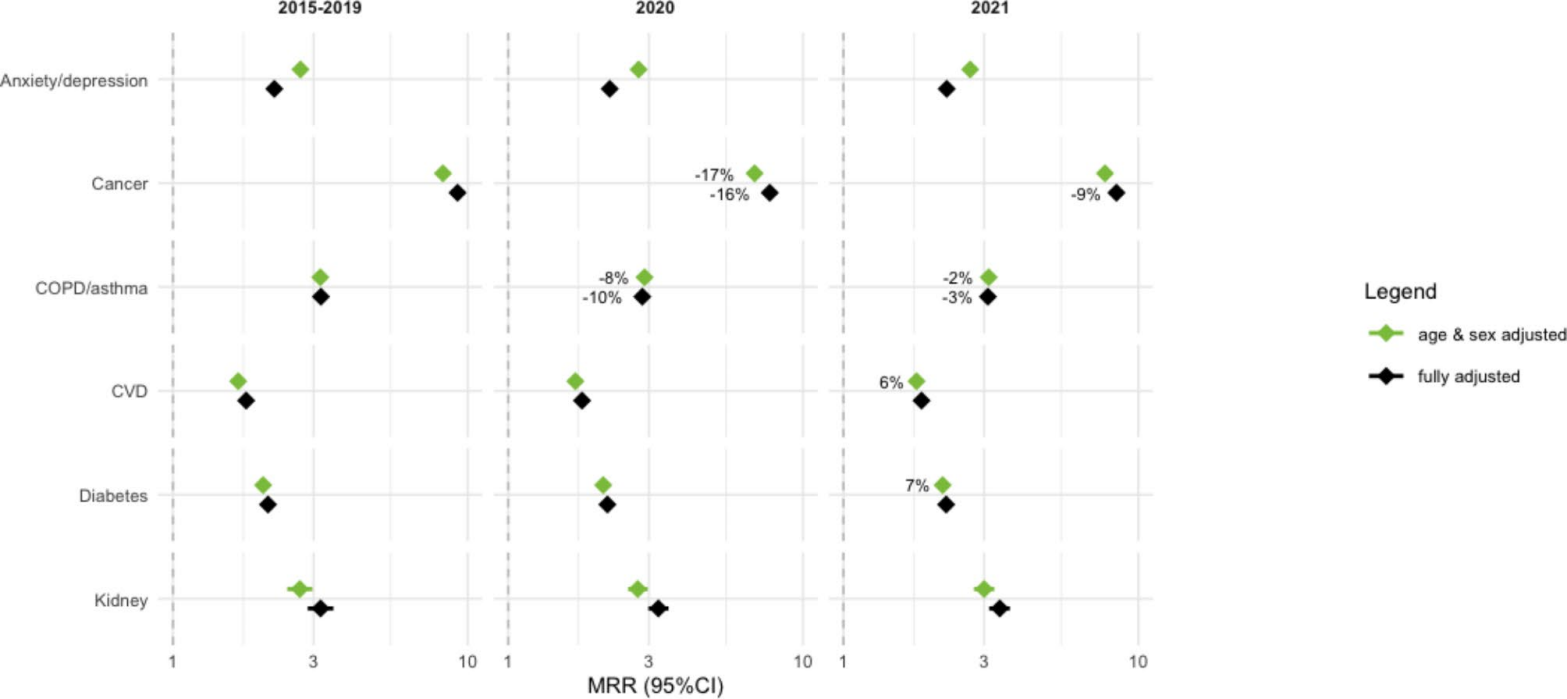
Mortality Rate Ratios (MRR) for six specific diseases, compared to persons with no identified disease. Note: Percentages mark significant excess or deficit mortality compared to the corresponding model for reference period 2015–2019. Green diamond represents the age-and sex-adjusted MRRs. Black diamonds represent the fully-adjusted MRRs. Overview available in Supplementary table S6

Figure 1 presents the MRRs and 95% confidence intervals for the specific disease under investigation compared to the reference category of persons with no identified disease. Age- and sex-adjusted MRRs and fully adjusted MRRs for the years 2020 and 2021 were compared to the reference period 2015–2019.

Significant mortality deficits were found for cancer and asthma or COPD, diseases already showing large relative mortality differences in the reference period 2015–2019. Persons with health care use related to cancer in the previous year experienced 8 to 9 times higher mortality compared with persons with no identified diseases during the reference period. In 2020, MRRs for cancer patients were approximately 16% lower compared to the reference period. In 2021, the fully adjusted MRRs still showed a significant mortality deficit of 9%. Relative mortality differences were also high for persons identified as asthma or COPD patients. During the reference period, mortality among this group was threefold that of persons with no identified diseases. During 2020, MRRs for asthma or COPD patients were 8–10% lower compared to the 2015–2019 period. A significant mortality deficit was still found in 2021 with 2–3% lower mortality compared to the reference periods.

Excess mortality was found for two groups: persons with cardiovascular diseases and diabetes in the previous year. The age-and sex-adjusted MRRs show significant excess mortality for CVD and diabetes patients of 6% and 7%, respectively, in 2021. The results for 2020 and for the fully adjusted model in 2021 were not significant.

Like in the analyses on the number of comorbidities, age and sex are key explanatory variables. Nonetheless, the full model with sociodemographic and socioeconomic variables explains the variability in mortality rates across different subgroups more effectively, as can be concluded from the Bayesian Information Criterion and pseudo-R-squared (Supplementary table S5). Generally, larger relative mortality inequalities are found for cancer and kidney disease when reviewing the fully adjusted MRRs compared to the age- and sex-adjusted MRRs. Contrary, for anxiety or depression, age- and sex-adjusted MRRs showed larger relative mortality inequalities compared to the fully adjusted estimates. These contrasting patterns observed for different health backgrounds suggest that these specific diseases have a complex association with the included sociodemographic and socioeconomic indicators. Not adjusting for these factors may underestimate or overestimate mortality disparities associated with these diseases.

## Discussion

The current study confirms the well-established observation that persons with pre-existing health conditions experience higher mortality than healthy individuals [36, 37]. Most strikingly, the study offers new insights into the significant changes in the mortality patterns of persons with pre-existing health conditions over the course of the COVID-19 crisis.

When considering absolute mortality inequalities, persons with and without poor pre-existing health faced a significant excess mortality in 2020. For example, women with no pre-existing disease experienced 11% more deaths in 2020 compared to the reference period. Excess mortality for women with pre-existing diseases in 2020 ranged between 7% (2 diseases) and 24% (5 diseases or more). In 2021, specific subgroups experienced a significant mortality deficit, suggesting a mortality displacement effect. This phenomenon occurs when deaths that would have happened anyway over time are accelerated by event (e.g., a disease outbreak, heatwave) [38, 39]. When considering relative mortality inequalities, no significant change in MRRs was observed during 2020 or 2021 in comparison with the reference period.

The analyses of specific pre-existing diseases show heterogeneous results. For *anxiety or depression*, the DSMRs indicate that both men and women suffering from these mental health conditions experienced significantly more deaths in 2020 and significantly fewer deaths in 2021, compared with the reference period. This can be potentially explained by a mortality displacement effect, as previous research has established a direct causal relationship between poor mental health and suicide and substance use-related causes of death [40, 41]. Elevated levels of anxiety and depression have been reported for Belgium during the first 15 months of the epidemic, especially during times of increased COVID-19 preventative measures (e.g., lockdown) [10]. Despite indications of worsening mental health during the COVID-19 period in Belgium, mortality trends for suicide in 2020 and 2021 strongly resemble 2019 levels, except for a decline among Belgian men in 2021 [42]. Another explanation may lie in the association between anxiety and depressive symptoms and the engagement to follow COVID-19 preventive behaviours, thus increasing the potential for elevated COVID-19 mortality [43].

For *cardiovascular diseases and diabetes*, significant excess mortality was observed for both men and women in 2020, but no significant change with the reference period was observed for 2021. Both disease groups have been known to have a complex, bidirectional relationship with COVID-19. On the one hand, cardiovascular diseases and diabetes have been identified as important risk factors for adverse COVID-19 health outcomes [23, 44, 45]. On the other hand, COVID-19 complications have been associated with additional cardiovascular diseases [46], and COVID-19 treatments with worsening diabetes [47]. Previous research has revealed that the COVID-19 crisis has led to delays in seeking medical help, delayed care and increased mortality for cardiovascular and diabetes patients [48–50]. According to a widescale hospital audit during COVID-times in Belgium, the delays in cardiology wards had been almost completely reduced by May 2021 [51]. Diabetes treatment centres also seemed to have experienced considerable disruptions, but there are indications that their services had stabilised by September 2020 [52]. In addition, telehealth opportunities were developed early on in the pandemic for diabetes patients [53]. It is possible that these efforts, combined with increasing knowledge on effective COVID-treatments with regard to multimorbidity, led to similar mortality patterns in 2021 for theses disease groups than in the reference period.

Although *cancer* has also been identified as a risk factor for COVID-19 mortality [44, 45], the DSMRs show mortality deficits for men and women with cancer in 2020 and for women with cancer in 2021. The MRRs further present mortality deficits in 2020 and 2021 for persons with cancer. Potential explanations can be found in the wider disease context, including prioritisation of certain vulnerable disease groups in testing, treatment and preventive care measures, and the self-protective behaviour of certain patient groups during the pandemic. But, interestingly, our results differ from a previously published mortality study of a nationwide cohort of Belgian cancer patients, revealing excess mortality during the first half of 2020 after which mortality rates restored to normal levels [54]. While sensitivity analyses demonstrate a comparable sex and age distribution to the general Belgian population, it is possible that our study population differs from the general cancer patient population. By the end of 2020, over 5,000 fewer cancer diagnoses were reported in Belgium than expected, with significant drops in early-stage diagnoses for several cancer types and higher prevalence of advanced stages [55]. This may lead to an underestimation of the 2021 excess mortality pattern for persons with cancer. A future study should focus on causes of death and the role of pre-existing health, as there is some evidence for increased hospital treatments and decreased mortality for some specific cancer types (e.g., prostate cancer) during the early phases of the COVID-19 crisis [13, 54–56].

The findings for *asthma and COPD* are also noteworthy as both men and women with these diseases experienced a significant mortality deficit in 2021 according to the DSMRs. In addition, the MRRs show significant mortality deficits in 2020 and 2021. Mixed results for these conditions been reported previously [23, 45, 57]. In addition to explanations related to targeted care and prevention measures for this patient group, the available data may provide an incomplete view on asthma and COPD patients. According to recent assessments of the use of health insurance data for defining chronic diseases, the method provides sound results, except for asthma and COPD, where low sensitivity impedes accurate identification [58, 59]. Berete and colleagues note that not all people with asthma or COPD take their medication or perhaps below the threshold used in the diseases group definition [58]. It is possible that the operationalisation used in this article provides a select group of persons with more severe symptoms and/or persons who diligently follow a medication regiment. It is likely that these people would display self-protective behaviour during the pandemic.

Lastly, the mortality pattern of patients with *chronic kidney disease* closely resembled that of pre-COVID times, despite previous studies indicating increased mortality risks for this group [23, 60].

Pre-existing health, age and sex are key to understanding patterns of excess and deficit mortality. Nonetheless, our study underlines the importance of including sociodemographic and socioeconomic information to effectively explain mortality differences. The comparison between the model with only adjustment for age and sex and the fully adjusted model revealed the potential for underestimation (in the case of cancer and chronic kidney disease) and overestimation (in the case of anxiety of depression) of MRRs by disease group when not taking sociodemographic and socioeconomic factors into account. In order to investigate individual-level pre-existing health information and social background characteristics simultaneously, further efforts need to be made to expand data collection, open up existing data sources and improve the interoperability between data sources. A better understanding of the sociodemographic and socioeconomic patterning of pre-existing health problems and mortality is crucial to identify the mechanisms behind the complex causal relationship between social inequalities, multimorbidity and mortality [61].

The major strengths of this study include the availability of individual-level information on pre-existing health situation and social background for a large population-based study population. Our study has four limitations. First, our study population may not be representative for the general Belgian population. The sensitivity analyses suggest a similar age and sex structure as the Belgian population, but a higher proportion of Solidaris members are entitled to increased reimbursement compared to the general population in Belgium. It remains unclear to which extent the study population reflects the general distribution of pre-existing health problems. When comparing our results with the mortality patterns in the general population, we find similar patterns, although at lower levels. For example, male Solidaris members experienced 1,486 deaths per 100,000 representing an excess mortality of 10% in 2020. Using mortality data from statistics Belgium, we calculated that Belgian men experienced 2,284 deaths per 100,000 also representing an excess mortality of 10% in 2020 compared with 2015–2019. Second, pre-existing health was defined using reimbursement information, which is not the same as diagnostic information. As mentioned previously for asthma and COPD, a group of patients may have been overlooked. To date, the validity of this method was corroborated for diabetes and cardiovascular diseases, but, to our knowledge, no such investigation was performed for cancer, anxiety or depression or chronic kidney disease [58]. Specifically, during the COVID-19 period, changes in the health seeking behaviour and disruptions in the health care system in 2020 may have led to incomplete or inaccurate registration of conditions, potentially misclassifying disease groups defined in 2021 [12, 51, 55, 62]. As a result, the reference group for the analyses on the relative mortality inequalities may have contained more undiagnosed and untreated individuals in 2021, potentially making the diagnosed cases more selective in terms of disease severity and progression. A comprehensive understanding of the impact of undiagnosed and untreated conditions is essential for accurately assessing excess mortality patterns and guiding future health policies and interventions to ensure more equitable healthcare responses during crises.

Third, our dataset lacked information on vaccination coverage a key factor known to reduce COVID-19 mortality in immunised patients [63]. Persons with at least one pre-existing health condition achieved a higher and earlier vaccination plateau compared to those without underlying condition [63]. Yet, vaccination rates were lower in socially vulnerable groups [64]. Future studies should therefore explore the complex interplay between vaccination coverage, comorbidities, social vulnerabilities and their combined impact on mortality. Fourth, previous research has highlighted the significance of heatwaves in excess mortality patterns, such as a notable heatwave in the summer of 2021 [62, 63]. The current study did not include information on temperature and was, therefore, not able to disentangle the complex interplay between external environmental factors, pre-existing health conditions, and social background characteristics.

In conclusion, the current study used individual-level information from the health insurance fund Solidaris to better understand the drivers of excess mortality during the COVID-19 crisis. Considering the pre-existing health situation is crucial, given the diverse mortality patterns observed during the COVID-19 crisis with excess and deficit mortality observed for specific disease groups. Some evidence is found of mortality displacement effects among persons suffering from anxiety or depression, suggesting a complex interplay between the pandemic and already existing social health inequalities. Furthermore, the observed patterns for relative mortality inequalities indicate mortality deficits for cancer and asthma or COPD in 2020 and 2021, highlighting the importance of considering the wider health care context and self-protective behaviours of vulnerable patient groups. Given the association between sex and gender and both pre-existing health conditions, mortality and social factors, future research should delve deeper into these complex pathways, e.g., by applying more advanced causal inference techniques. Our analyses further demonstrate the importance of accounting for sociodemographic and socioeconomic factor in excess mortality studies. A more comprehensive understanding of the links between social inequalities, (multi)morbidity and mortality are needed to develop more precise crisis response interventions and policies. Addressing social health inequalities and improving chronic disease management can enhance the resilience of healthcare systems, protect vulnerable populations, and improve overall health outcomes in both crisis and non-crisis times.

## Electronic supplementary material

Below is the link to the electronic supplementary material.

Supplementary Material 1

## Data Availability

The analyses are based on data Solidaris and cannot be made available due to strict privacy regulations.

## Abbreviations

ATC: Anatomical, Therapeutic, and Chemical codes
CI: 95% Confidence Interval
COPD: Chronic Obstructive Pulmonary Disease
CVD: Cardiovascular Diseases
DSMR: Directly Standardised Mortality Rates
MRR: Mortality Rate Ratios
RIZIV-INAMI: National Institute for Health and Disability Insurance
